# Supplementary material for: Association of remoteness and ethnicity with major amputation following minor amputation to treat diabetes-related foot disease
Source: PLoS One. 2024 Jul 5;19(7):e0302186. doi: 10.1371/journal.pone.0302186 (PMC11226033; doi:10.1371/journal.pone.0302186)
Supplement: S1 Table — (DOCX) [file pone.0302186.s001.docx]

S1 Table: Association of remoteness with major amputation following a minor amputation to treat diabetes-related foot disease

| Risk factor | Univariate analysis HR [95% CI] | P value | Multivariate analysis | | Likelihood ratio test statistic |
| --- | --- | --- | --- | --- | --- |
| Age | 1.00 [0.99-1.02] | 0.624 | 0.99 [0.98-1.01] | 0.668 | NA |
| Sex | 1.14 [0.76-1.78] | 0.540 | 1.16 [0.76-1.75] | 0.492 | 0.910 |
| Smoking | 1.05 [0.71-1.55] | 0.797 | 0.89 [0.60-1.31] | 0.549 | 0.294 |
| IHD | 2.15 [1.46-3.17] | **<0.001** | 1.81 [1.19-2.74] | **0.005** | 29.484 |
| PAD | 2.13 [1.45-3.13] | **<0.001** | 2.53 [1.69-3.78] | **<0.001** | 21.694 |
| ESRF | 1.85 [1.07-3.20] | **0.029** | 1.37 [0.78-2.41] | 0.276 | 2.642 |
| Osteomyelitis | 2.61 [1.77-3.851] | **<0.001** | 2.83 [1.90-4.23] | **<0.001** | 54.518 |
| Ulcer | 4.71 [2.06-10.77] | **<0.001** | 5.15 [2.25-11.81] | **<0.001** | 48.76 |
| Remoteness | 0.97 [0.67-1.47] | 0.966 | 0.96 [0.64-1.42] | 0.826 | 2.515 |

IHD; ischemic heart disease, PAD; peripheral artery disease, ESRF; end stage renal failure, NA; not applicable. Bold indicates significant results
