# Supplementary material for: Association of remoteness and ethnicity with major amputation following minor amputation to treat diabetes-related foot disease
Source: PLoS One. 2024 Jul 5;19(7):e0302186. doi: 10.1371/journal.pone.0302186 (PMC11226033; doi:10.1371/journal.pone.0302186)
Supplement: S2 Table — (DOCX) [file pone.0302186.s002.docx]

S2 Table: Contribution of individual risk factors for the overall cox regression model that included remoteness

| Risk factor removed at each step | -2 Log Likelihood value of each model | Log Likelihood ratio following comparison with the previous model | Risk factor contribution: significant or not |
| --- | --- | --- | --- |
| Model with all factors | 1141.766 |  |  |
| Residency status | 1141.814 | 0.096 | Not significant |
| Ulcer | 1166.194 | 48.76 | **Significant** |
| Osteomyelitis | 1193.453 | 54.518 | **Significant** |
| ESRF | 1194.774 | 2.642 | Not significant |
| PAD | 1205.621 | 21.694 | **Significant** |
| IHD | 1220.363 | 29.484 | **Significant** |
| Smoking | 1220.510 | 0.294 | Not significant |
| Sex | 1220.965 | 0.910 | Not significant |
| Age | NA |  |  |

Note: According to the Chi-square distribution table, the critical value for distribution of one degree of freedom is 3.84, which is the significance level. After removal of one factor from the model if the Log Likelihood ratio value becomes higher than the critical value of 3.84, that particular factor is considered to have contribute significantly to the overall model.

IHD; ischemic heart disease, PAD; peripheral artery disease, ESRF; end stage renal failure, NA; not applicable
