# Supplementary material for: Association of remoteness and ethnicity with major amputation following minor amputation to treat diabetes-related foot disease
Source: PLoS One. 2024 Jul 5;19(7):e0302186. doi: 10.1371/journal.pone.0302186 (PMC11226033; doi:10.1371/journal.pone.0302186)
Supplement: S4 Table — (DOCX) [file pone.0302186.s004.docx]

S4 Table: Association of ethnicity with major amputation following a minor amputation to treat diabetes-related foot disease.

| Risk factor | Univariate analysis, HR [95% CI] | P value | Multivariate analysis | | Likelihood ratio test statistic |
| --- | --- | --- | --- | --- | --- |
| Age | 1.00 [0.99-1.02] | 0.624 | 1.00 [0.98-1.02] | 0.917 | NA |
| Sex | 1.14 [0.76-1.78] | 0.540 | 1.10 [0.72-1.69] | 0.650 | 0.910 |
| Smoking | 1.05 [0.71-1.55] | 0.797 | 0.89 [0.60-1.32] | 0.567 | 0.294 |
| IHD | 2.15 [1.46-3.17] | **<0.001** | 1.83 [1.21-2.77] | **0.005** | 29.484 |
| PAD | 2.13 [1.45-3.13] | **<0.001** | 2.51 [1.68-3.76] | **<0.001** | 21.694 |
| ESRF | 1.85 [1.07-3.20] | **0.029** | 1.35 [0.77-2.38] | 0.298 | 2.642 |
| Osteomyelitis | 2.61 [1.77-3.851] | **<0.001** | 2.79 [1.87-4.16] | **<0.001** | 54.518 |
| Ulcer | 4.71 [2.06-10.77] | **<0.001** | 5.02 [2.19-11.51 | **<0.001** | 48.76 |
| Aboriginal and Torres Strait Islander status | 1.44 [0.96-2.16] | 0.078 | 1.29 [0.83-2.00] | 0.258 | 0.096 |

IHD; ischemic heart disease, PAD; peripheral artery disease, ESRF; end stage renal failure, NA; not applicable. Bold indicates significant results
