# Supplementary material for: Association of remoteness and ethnicity with major amputation following minor amputation to treat diabetes-related foot disease
Source: PLoS One. 2024 Jul 5;19(7):e0302186. doi: 10.1371/journal.pone.0302186 (PMC11226033; doi:10.1371/journal.pone.0302186)
Supplement: S5 Table — (DOCX) [file pone.0302186.s005.docx]

S5 Table: C statistic or the area under the curve of receiver operating characteristic curves for each risk factor and its ability to predict the outcome of a major amputation among participants who underwent a minor amputation following diabetes-related foot disease.

| Risk factor | c-index [95% confident intervals] | P value | Predictive ability |
| --- | --- | --- | --- |
| Age | 0.494 [0.431-0.558] | 0.860 | failed |
| Sex | 0.531 [0.467-0.595] | 0.343 | failed |
| Smoking | 0.495 [0.432-0.559] | 0.884 | failed |
| IHD | 0.601 [0.539-0.664] | **0.002** | poor |
| PAD | 0.601 [0.537-0.664] | **0.002** | poor |
| ESRF | 0.526 [0.461-0.590] | 0.429 | failed |
| Osteomyelitis | 0.614 [0.549-0.678] | **<0.001** | poor |
| Ulcer | 0.577 [0.519-0.635] | **0.017** | failed |
| Residency status | 0.505 [0.442-0.569] | 0.871 | failed |
| Aboriginal and Torres Strait Islander Status | 0.541 [0.477-0.606] | 0.204 | failed |

Note: Predictive ability of a risk factor based on the area under the curve: 0.9-1; excellent, 0.8-0.9; good, 0.7-0.8; fair, 0.6-0.7; poor and <0.6; failed.

Bold indicates significant results. IHD; ischemic heart disease, PAD; peripheral artery disease, ESRF; end stage renal failure
