# Supplementary material for: Association of remoteness and ethnicity with major amputation following minor amputation to treat diabetes-related foot disease
Source: PLoS One. 2024 Jul 5;19(7):e0302186. doi: 10.1371/journal.pone.0302186 (PMC11226033; doi:10.1371/journal.pone.0302186)
Supplement: S6 Table — (DOCX) [file pone.0302186.s006.docx]

S6 Table: Cox proportional hazard analyses for the association between Modified Monash Model (MMC) categories and Aboriginal and Torres Strait Islander status versus repeat minor amputation and death.

| Risk factor | Unadjusted hazard ratio [95% CI] | p-value | Adjusted hazard ratio [95% CI] ^a^ | p-value | | Adjusted hazard ratio [95% CI] ^b^ | p-value |
| --- | --- | --- | --- | --- | --- | --- | --- |
| **Minor Amputations** | | | | | | | |
| MMM category | 1.03 [0.87-1.22] | 0.764 | 1.02 [0.86-1.210 | 0.797 |  | |  |
| Aboriginal and Torres Strait Islander status | 1.09 [0.82-1.46] | 0.549 |  |  | 1.10 [0.81-1.50] | | 0.549 |
| Age | 1.00 [0.99-1.01] | 0.777 | 1.00 [0.99-1.01] | 0.543 | 0.99 [0.99-1.01] | | 0.683 |
| Sex | 0.85 [0.64-1.13] | 0.263 | 0.85 [0.63-1.13] | 0.262 | 0.84 [0.62-1.22] | | 0.234 |
| Smoking | 1.18 [0.91-1.52] | 0.224 | 1.11 [0.84-1.45] | 0.466 | 1.10 [0.84-1.44] | | 0.501 |
| PAD | 1.23 [0.94-1.60] | 0.134 | 1.22 [0.93-1.61] | 0.159 | 1.22 [0.93-1.62] | | 0.156 |
| ESRF | 1.00 [0.63-1.61] | 0.990 | 0.99 [0.61-1.59] | 0.952 | 0.97 [0.60-1.57] | | 0.896 |
| **Death** | | | | | | | |
| MMM category | 0.99 [ 0.84-1.15] | 0.860 | 0.97 [0.83-1.14] | 0.715 |  | |  |
| Aboriginal and Torres Strait Islander status | 0.89 [0.67-1.18] | 0.415 |  |  | 1.01 [0.75-1.38] | | 0.929 |
| Age | 1.01 [0.99-1.02] | 0.101 | 1.01 [0.99-1.02] | 0.336 | 1.01 [0.99-1.02] | | 0.353 |
| Sex | 0.81 [0.61-1.06] | 0.123 | 0.81 [0.61-1.08] | 0.145 | 0.81 [0.61-1.08] | | 0.148 |
| Smoking | 0.86 [0.67-1.10] | 0.227 | 0.80 [0.62-1.03] | 0.087 | 0.80 [0.62-1.03] | | 0.087 |
| PAD | 1.14 [0.88-1.47] | 0.323 | 1.22 [0.93-1.06] | 0.153 | 1.22 [0.93-1.60] | | 0.153 |
| ESRF | 0.71 [0.46-1.09] | 0.119 | 0.67 [0.83-1.14] | 0.715 | 0.67 [0.42-1.05] | | 0.081 |

Bold indicates significant results. MMC; Modified Monash Classification, PAD; peripheral artery disease, ESRF; end stage renal failure

^a^ Adjusted for MMM category, age, sex, smoking, PAD and ESRF

^b^ Adjusted for Aboriginal and Torres Strait Islander Status, age, sex, smoking, PAD and ESRF.
