# Supplementary material for: Association of remoteness and ethnicity with major amputation following minor amputation to treat diabetes-related foot disease
Source: PLoS One. 2024 Jul 5;19(7):e0302186. doi: 10.1371/journal.pone.0302186 (PMC11226033; doi:10.1371/journal.pone.0302186)
Supplement: Attachment — Submitted filename: Response to Reviwers 27032024.docx [file pone.0302186.s009.docx]

27-03-2024

Dr Yee Gary Ang
Academic Editor
PLOS ONE

Title of the manuscript:

PONE-D-23-28544R1

**Association of remoteness and ethnicity with major amputation following minor amputation to treat diabetes-related foot disease.**

PLOS ONE

Thank you for accepting this manuscript for publication. We wish to resubmit this manuscript after making the changes the reviewers suggested. The detail comments are shown below.

We hope to hear from you soon.

Disclosure: The funders had no role in study design, data collection and analysis, decision to publish, or preparation of the manuscript.

Thank you.

Professor Jonathan Golledge.

**Please kindly address some of the comment although both reviewers have recommended acceptance.**

1) The research methodology is fundamentally solid, but a more detailed exposition of the recruitment process would enhance the paper's clarity and robustness.

Thank you for the comment.

The “Population” section of our Materials and Methods has now been changed to read as follows.

## **Population**

All patients were identified through Operating Rooms Management System (ORMIS); a system that records all surgical procedures conducted in TUH. For inclusion, participants had to have undergone a minor amputation (i.e. an amputation distal to the ankle joint [3]) as treatment for DFD during the study period. Participants also had to be previously diagnosed with diabetes and have data available on place of residence and Aboriginal and Torres Strait Islander ethnicity. The first minor amputation that occurred during the study period between 1^st^ of January 2000 to 31^st^ of December 2019 was defined as the index amputation. Patients with previous major amputation (unilateral or bilateral), those who underwent an amputation procedure in the absence of diabetes, those who were <18 years of age and those with missing patient records were excluded. After identification of the participant through ORMIS, the relevant participant’s hospital admission record during which the participant had the index minor amputation procedure was accessed to obtain the necessary data. Subsequent records were also obtained to identify further outcomes.

Minor amputation was defined as amputation distal to the ankle joint [3]. Toe amputations were defined as amputation of a toe at or distal to the metatarso-phalangeal joint. A trans-metatarsal amputation was defined as an amputation across metatarsal bones. Forefoot amputation was defined as an amputation distal to the ankle joint at or proximal to tarso-metatarsal joints [3]. Participants were identified using ORMIS as previously described [16]. All patients undergoing any amputation procedure in the TUH are recorded in this system.

2) Certain sections, particularly the Abstract, exhibit a lack of cohesion and fail to articulate a compelling argument for the study's significance.

Thank you for the Comment. The abstract has been changed to read as follows.

**Abstract**

**Introduction**

Minor amputation is commonly needed to treat diabetes-related foot disease (DFD). Remoteness of residence is known to limit access to healthcare and has previously been associated with poor outcomes. The primary aim of this study was to examine the associations between ethnicity and remoteness of residency with the risk of major amputation and death following initial treatment of DFD by minor amputation. A secondary aim was to identify risk factors for major amputation and death following minor amputation to treat DFD.

**Research design and methods.**

This was a retrospective analysis of data from patients who required a minor amputation to treat DFD between 2000 and 2019 at a regional tertiary hospital in Queensland, Australia. Baseline characteristics were collected together with remoteness of residence and ethnicity. Remoteness was classified according to the 2019 Modified Monash Model (MMM) system. Ethnicity was based on self-identification as an Aboriginal and Torres Strait Islander or non-Indigenous person. The outcomes of major amputation, repeat minor amputation and death were examined using Cox-proportional hazard analyses.

**Results**

A total of 534 participants were included, with 306 (57.3%) residing in metropolitan or regional centres, 228 (42.7%) in rural and remote communities and 144 (27.0%) were Aboriginal or Torres Strait Islander people. During a median (inter quartile range) follow-up of 4.0 (2.1-7.6) years, 103 participants (19.3%) had major amputation, 230 (43.1%) had repeat minor amputation and 250 (46.8%) died. The risks (hazard ratio [95% CI]) of major amputation and death were not significantly higher in participants residing in rural and remote areas (0.97, 0.67-1.47; and 0.98, 0.76-1.26) or in Aboriginal or Torres Strait Islander people (HR 1.44, 95% CI 0.96, 2.16 and HR 0.89, 95% CI 0.67, 1.18). Ischemic heart disease (IHD), peripheral artery disease (PAD), osteomyelitis and foot ulceration (p<0.001 in all instances) were independent risk factors for major amputation.

**Conclusion**

Major amputation and death are common following minor amputation to treat DFD and people with IHD, PAD and osteomyelitis have an increased risk of major amputation. Aboriginal and Torres Strait Islander People and residents of remote areas were not at excess risk of major amputation.

3) Moreover, the discussion of the findings, especially considering their divergence from previous research on Australian Indigenous populations with Diabetic Foot Disease (DFD), lacks depth. The paper does not sufficiently explore the implications of these findings for future funding, service access for remote populations, and the overall management of DFD in Indigenous communities.

The presentation of the findings appears somewhat cursory, with little consideration of their broader impact or how they might inform future clinical practices for DFD across all populations. This oversight suggests a missed opportunity to leverage the collected data for substantive clinical improvements.

Thank you for the comment. The revised discussion now reads as follows.

# Discussion

This study is the first to assess the impact of remoteness of residence on the outcome of people having minor amputation to treat DFD. Similar studies have been conducted in patients with other forms of vascular disease such as abdominal aortic aneurysms and peripheral artery disease but not in people with DFD [25]. The setting in North Queensland, Australia was well suited for this investigation because the population is widely dispersed with patients presenting from both regional and rural areas with considerable travel time to TUH [25]. This study included 534 participants with 27% identifying as Aboriginal and/or Torres Strait Islander People. One fifth of the participants underwent a major amputation and about half of participants died during a median follow-up of 4 years. Outcomes were not significantly different for Aboriginal and Torres Strait Islander and non-Indigenous people, which may reflect equivalent care given to participants presenting from diverse backgrounds [28]. This is in contrast to previous studies that showed poorer outcomes among Aboriginal and Torres Strait Islanders compared to non-Indigenous patients [18, 29]. IHD, PAD, presence of osteomyelitis and foot ulceration during the index minor amputation were independent predictors of major amputation, as previously reported in other studies [16, 17].

More than one million people worldwide undergo a lower extremity amputation each year to treat DFD [30]. Such amputations cause significant morbidity and mortality [8, 31]. A recent systematic review reported that global rates of hospital admission for all DFD conditions are considerably greater than those for amputations alone [32]. In the USA the rate of non-traumatic limb amputation increased by 50% between 2009 and 2015 [33]. A repeat amputation rate of 26% within 1-year of initial amputation was reported in a study of 71300 participants [31]. Despite the availability of studies investigating the overall rates of amputation [34], there have been no studies conducted to look at subsequent major amputation following an index minor amputation in Australia.

In the current study, during a median follow-up period of 4 years 47% of the participants died and 43% had a repeat minor amputation emphasising the high morbidity and mortality associated with DFD. DFD causes substantial social, psychological, and physical burden [35, 36]. Similar results have been reported in US populations [37]. This highlights the need for better secondary prevention, which is challenging to implement in patients presenting from vast geographic areas, especially in rural populations where access to health services is limited. In the current study participants with IHD, PAD, osteomyelitis and foot ulceration had an increased risk of major amputation, as has been previously reported from multiple studies [38, 39]. Patients with PAD were twice as likely to have a major amputation as compared to those with no PAD, similar to findings from previous studies [16, 21]. A previous study from North Queensland found an excess of distal tibial artery disease which can be challenging to revascularise [40]. The distal nature of the artery disease and the challenges of implementing medical management may have contributed to the high amputation rates reported [40]. This study found that ESRF was also an independent risk factor for major amputation, as previously reported [41]. The association of IHD with major amputation may be secondary to undetected PAD in these participants [42].

A number of previous studies have reported an excess of major amputations in rural locations [43]. In contrast, this study found that there is no significant difference in the rates of amputation in participants living in remote locations compared to those living in larger towns. This finding maybe reflective of state-wide expansion in podiatry care provides to rural locations, along with use of telehealth services for endocrinology introduced in Queensland [28].

The current study found that the rates of major amputation were not significantly different in non-Indigenous and Australian First Nations patients. The unadjusted HR did however suggest a non-significant (p=0.078) 1.44-fold excess risk of major amputation in Aboriginal and Torres Strait Islander compared to non-Indigenous patients. The lack of significant difference in outcomes may be reflect multiple health promotion programs that were implemented by the government to close the healthcare gap or may simply reflect insufficient sample size to adequately compare outcomes [43]. The findings of this study highlight the need for continued efforts to reduce the morbidity and mortality related to DFD, via frequent podiatry care and improved medical management. Use of telehealth services and community-based podiatry is recommended to improve access to services in rural localities [44]. Early referral of patients to tertiary care centres is recommended for early interventions to prevent major amputation by revascularisation procedures.

The current study has a number of strengths and limitations. Strengths included the relatively large sample size and the inclusion of substantial numbers of Aboriginal and Torres Strait Islander participants. Limitations include the retrospective study design and the lack of information on the Wound Ischaemia Foot Infection (WIFI) classification and use of revascularisation procedures. It is also possible that some outcome evets may have been missed if participants moved outside the North Queensland region. It is also important to note that these findings may not be generalisable to other regions where delivery of health care and composition of the population may be different.
